# Environmental suitability for lymphatic filariasis in Nigeria

**DOI:** 10.1186/s13071-018-3097-9

**Published:** 2018-09-17

**Authors:** Obiora A. Eneanya, Jorge Cano, Ilaria Dorigatti, Ifeoma Anagbogu, Chukwu Okoronkwo, Tini Garske, Christl A. Donnelly

**Affiliations:** 1 MRC Centre for Global Infectious Disease Analysis, Department of Infectious Disease Epidemiology, Imperial College London, London, UK; 2 Faculty of Infectious and Tropical Diseases, London School of Hygiene and Tropical Medicine, London, UK; 3 Federal Ministry of Health, Abuja, Nigeria; 4 Department of Statistics, University of Oxford, Oxford, UK

**Keywords:** Lymphatic filariasis, Ensemble modelling, Machine learning, Generalised boosted model (GBM), Random forest (RF)

## Abstract

**Background:**

Lymphatic filariasis (LF) is a mosquito-borne parasitic disease and a major cause of disability worldwide. It is one of the neglected tropical diseases identified by the World Health Organization for elimination as a public health problem by 2020. Maps displaying disease distribution are helpful tools to identify high-risk areas and target scarce control resources.

**Methods:**

We used pre-intervention site-level occurrence data from 1192 survey sites collected during extensive mapping surveys by the Nigeria Ministry of Health. Using an ensemble of machine learning modelling algorithms (generalised boosted models and random forest), we mapped the ecological niche of LF at a spatial resolution of 1 km^2^. By overlaying gridded estimates of population density, we estimated the human population living in LF risk areas on a 100 × 100 m scale.

**Results:**

Our maps demonstrate that there is a heterogeneous distribution of LF risk areas across Nigeria, with large portions of northern Nigeria having more environmentally suitable conditions for the occurrence of LF. Here we estimated that approximately 110 million individuals live in areas at risk of LF transmission.

**Conclusions:**

Machine learning and ensemble modelling are powerful tools to map disease risk and are known to yield more accurate predictive models with less uncertainty than single models. The resulting map provides a geographical framework to target control efforts and assess its potential impacts.

## Background

Lymphatic filariasis (LF) is a mosquito-borne disease endemic in tropical regions and caused by the parasitic nematode *Wuchereria bancrofti* in Africa, and by *Brugia malayi* and *B. timori* in Southeast Asia [1]*.* These parasites are transmitted by various species of mosquitoes, with *Anopheles* spp. being major vectors in Africa [1, 2]. Other mosquito species of the genera *Culex* and *Mansonia* also contribute to transmission to some extent, particularly in urban and peri-urban settings [3, 4]. The majority of infected individuals are asymptomatic, but infections can lead to lymphedema, hydrocele and swellings of the breasts in women [5]. An independent International Task Force for Disease Eradication included LF as one of the nine diseases targeted for elimination [6], and in 1997 the World Health Assembly adopted Resolution WHA50.29 embarking on a global campaign to eliminate LF. Elimination of LF as a public health problem is deemed feasible for a number of reasons: (i) mosquitoes are very inefficient transmitters of filarial parasites [7]; (ii) the small number of animal reservoirs are restricted to particular foci for *B. malayi*, and there are no animal reservoirs for *W. bancrofti* [8]; and (iii) the availability of improved diagnostic tools and the existence of practical interventions for interruption of transmission [9–11]*.*

An understanding of the geographical distribution of LF is required to underpin national elimination programmes. This enables more effective targeting of control efforts on highly endemic areas. Early maps of disease distribution have mostly relied on field surveys at national or sub-national levels [12–15], often with entire administrative units classified based on disease prevalence with no account for within-unit heterogeneity [16]. This might be useful for roughly estimating disease burden [17, 18]; however, such estimates fail to accurately represent disease burden and highly endemic foci may be misclassified [18]. To account for within-region heterogeneity, maps have been created by applying geostatistical modelling on point prevalence data, in combination with potential disease drivers (i.e. climatic, environmental and demographic factors), due to their impacts on mosquito populations and parasite biology [16, 19–24].

In 2003, the Nigerian Lymphatic Filariasis Elimination Programme (NLFEP) commenced LF mapping on a national scale, and to date, 761 out of 774 Local Government Areas (LGAs) have been mapped using immunochromatographic card tests (ICT) [15]. Of these, 574 LGAs are classed as endemic and targeted for mass drug administration (MDA), and 187 LGAs non-endemic for LF [15]. In total, an estimated 128 million people in Nigeria are thought to require preventive chemotherapy, and as of 2016, 54% of this population had been treated [25]. After more than five rounds of MDA in Plateau and Nassarawa states, Transmission Assessment Survey 1 (TAS-1) showed evidence of interruption of LF transmission in these areas [26]. However, for the vast areas of the country in which LF is present, understanding disease distribution on a finer scale is key for more focussed targeting of control measures.

In this work we aim to (i) describe and map the ecological niche of LF in Nigeria and (ii) estimate the human population living in areas that are environmentally suitable for disease transmission. Here we fitted seven different model classes to the same selection of training and evaluation data points, and projected the final ecological niche map using an ensemble of the two best performing models.

## Methods

### LF occurrence data

Data used for ecological niche modelling (ENM) were pre-intervention site-level data collected during mapping surveys conducted by the Nigeria Ministry of Health from 2000–2013. The sampling geographical level for LF mapping is the implementation unit (IU), which corresponds to a Local Government Area (LGA), the second level administrative unit in Nigeria. In total, we had 1192 data points covering all 36 states, and the Federal Capital Territory in Nigeria. A uniform survey methodology was applied to all survey locations and study participants were tested for the presence of filarial antigenemia using a point-care rapid test (immunochromatographic card test), and following the mapping protocol of the World Health Organization (WHO) Operational Guidelines for Mapping of Bancroftian Filariasis in Africa [27]. Briefly, within each IU (LGA), at least one sample village is randomly selected for survey. Selected villages must be located at least 50 km apart from each other. In each selected village, 50–100 adults (seeking an equal number of males and females when possible, and > 15 years of age) are tested. If 20% or more of the first 50 individuals tested result positive, testing is stopped, and the entire IU is recorded as positive for LF. Otherwise, testing is continued until 100 adults have been examined. In the end, IUs with at least one sampled village yielding a prevalence equal to or more than 1% are considered to be endemic for LF and subsequently targeted with MDA.

**Fig. 1:**
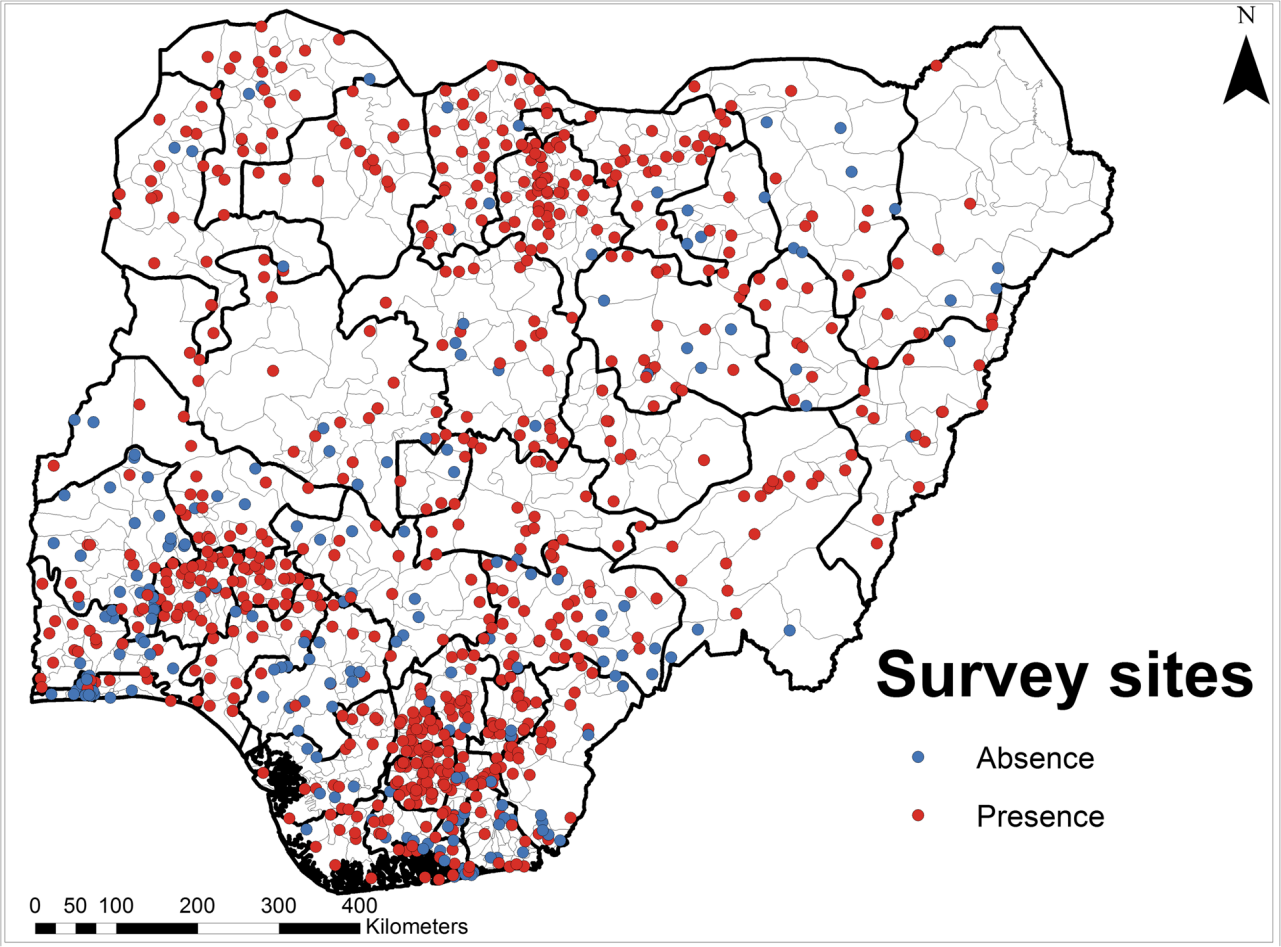
Location of study sites in Nigeria. Red points show sites with at least one LF case and blue points show sites with no LF case

For this analysis, occurrence (coded 1) was considered when at least one LF case was recorded during mapping surveys and absence (coded 0) when none of the individuals tested resulted positive to the LF rapid test [28]. In total we had 932 ‘presence’ and 260 ‘absence’ records for this modelling exercise. Figure 1 shows the distribution of survey points and presence-absence records used in this work.

### Climatic and environmental data

A suite of environmental variables was considered to describe the ecological niche of LF. Continuous gridded maps of climate, topography, vegetation and land use for Africa were obtained from different sources (Table 1). Climate variables related to precipitation and temperature were downloaded from the WorldClim database [29], which provides a set of global climate layers obtained by interpolation of the data for the period of 1950–2000 collected in weather stations distributed across the world. From the Consortium of Spatial Information (CGIAR-CSI) we obtained raster datasets of potential evapo-transpiration (PET), elevation and aridity index at 1 km^2^ resolution [30]. PET is a measure of the ability of the atmosphere to remove water through evapo-transpiration. Our elevation layer resulted from processing and resampling the gridded digital elevation models (DEM) derived from the 30-arcsecond DEM produced by the Shuttle Radar Topography Mission (SRTM) [31]. The elevation layer was used to generate two topography-related datasets: slope of terrain and flow accumulation.

**Table 1:** Environmental variables used in the ENM and their sources

Variables	Source
Annual cumulative precipitation	WorldClim [29]
Maximum temperature	
Mean temperature	
Minimum temperature	
Mean temperature of the coldest quarter	
Mean temperature of the warmest quarter	
Precipitation of the driest quarter	
Precipitation of the wettest quarter	
Potential evapo-transpiration (PET)	CGIAR-CSI [30]
Aridity index	
Elevation	SRTM [31]
Slope	Derived from elevation
Flow accumulation	Derived from slope
Distance to permanent rivers	Digital Global Chart [34]
Distance to nearest water bodies	Global Database of Lakes, Reservoirs and Wetlands [33]
Land surface temperature (LST)	AfSIS [35]
Enhanced vegetation index (EVI)	
Sand, silt, clay fractions	ISRIC [37]
Soil pH	
Major land cover (forest, agriculture, shrubland-grassland)	Arino et al [36]
Wetness index	Derived from slope and flow accumulation
Distance to stable lights 2006	Elvidge et al. [38]

To produce the flow accumulation layer, we initially created a flow direction layer in which the direction of flow was determined by the direction of the steepest descent from each cell in the elevation dataset. This was calculated as follows: change in elevation value/distance × 100. Flow accumulation was then calculated as the accumulated weight of all cells flowing into each downslope cell in the flow direction layer.

In addition, we calculated topographic wetness index (TWI) by applying the following algorithm

$$ TWI=\ln \left(a/ tan\beta \right), $$

where *a* is the upslope contributing area per unit contour length [or specific catchment area (SCA)], which can be approached by using the flow accumulation, and *β* is the local slope gradient for reflecting the local drainage potential [32].

We also produced continuous surfaces of straight-line distance (Euclidean distance) in kilometres to the nearest water body and permanent rivers. These data were derived from the Global Database of Lakes, Reservoirs and Wetlands [33] and Digital Global Chart [34], respectively. Raster datasets of averaged enhanced vegetation index (EVI) and land surface temperature (LST) for the period 2000–2015 were obtained from the African Soil Information System (AfSIS) project [35]. Here, EVI and LST were generated from remotely sensed data collected from the Moderate Resolution Imaging Spectoradiometer (MODIS) platform. MODIS collects earth data from the same place every 16 days at 250 m spatial resolution. Land cover types (according to the United Nations land cover classification system) were extracted from the GlobCover project at the European Space Agency [36]. Here, maps are derived by an automatic and regionally-tuned classification of a 300 m medium resolution imaging spectrometer (MERIS) sensor on board the ENVISAT satellite mission. Soil data (sand, silt and clay fractions, and soil pH) were downloaded from the International Soil Reference and Information Centre (ISRIC) project [37] at a spatial resolution of 250 m.

Finally, night-light (NL) emissivity for 2006 captured by the Operational Linescan System instrument on board a satellite of the Defence Meteorological Satellite Programme was used as a proxy measure of poverty across Nigeria [38]. This instrument measures visible and infrared radiation emitted at night-time, resulting in remote imagery of lights on the ground. This information has been correlated with gross domestic product in developed countries [39, 40] and, although far from being precise, could provide an indirect measure of poverty in developing countries [41]. NL emissivity is provided as gridded maps of 1 km^2^ resolution, and values that go from 0 (undetectable NL emissivity) to 60 (maximum NL emissivity). Alternatively, we estimated the Euclidean distance to stable night-lights, considered as NL values > 0.

Input grids were resampled to a common spatial resolution of 1 km^2^ using the nearest-neighbour approach [42], clipped to match the geographical extent of a map of mainland Nigeria, and aligned to it. Raster manipulation and processing were undertaken using *raster* package in R (v.3.3.2) [43]. All environmental covariates considered in our models are known to be biologically plausible for LF occurrence [16, 23].

### Model implementation

#### Selection of covariates

Covariate data were extracted corresponding to each of the presence-absence data points. In this work we explored the existence of multicollinearity using the variance inflation factor (VIF). Multicollinearity often arises in statistical models, and occurs when two or more covariates are not statistically independent leading to unstable estimates of variances of regression coefficients [44]. The VIF represents the amount of variability of a covariate which is explained by other covariates. For instance, the VIF for the *i*th covariate can be calculated as: VIF_*i*_ = 1/(1 – R^2^_*i*_), where R^2^_*i*_ is the coefficient of determination obtained by fitting a linear regression model for the *i*th independent covariate. The VIF of the suite of environmental covariates tested here was calculated and correlated variables were excluded in a stepwise procedure at a generally accepted threshold value of 10 [44]. Of the 24 covariates initially tested for multicollinearity, seven (average precipitation, mean temperature, average maximum temperature, average minimum temperature, aridity index, PET and sand soil type) were excluded from further analysis. All remaining covariates were considered to be independent and were included in the analysis. The multicollinearity test was implemented using the *usdm* package in R (v.3.3.2) [43].

The relative importance of the covariates to our presence-absence dataset was identified using the boosted regression trees (BRT) machine-learning algorithm. BRT is a combination of two algorithms, regression trees and boosting. This produces an additive regression model in which simple trees are fitted in a forward, stepwise fashion. This method has been widely used in disease prediction and considered a powerful tool for ecological studies [24, 45, 46]. Relative importance is defined as the frequency of selection of covariates for splitting, weighted by the squared improvements to the model, and averaged over all trees [45]. Higher relative importance scores, which are computed as percentages (and scaled to a maximum sum of 100%), indicate greater contribution to the model. Variables that showed no substantial contribution to the model (we set this at a threshold of 10%) [47] were excluded in fitting the final ensemble of models. Variables dropped at this stage were soil pH, night-light emissivity, EVI, distance to the nearest water body, distance to rivers, flow accumulation, mean temperature of the coldest quarter, mean temperature of the warmest quarter, and clay and silt soil fraction. The remaining predictors: precipitation in the driest quarter, precipitation in the wettest quarter, wetness index, land surface temperature, elevation, distance to stable lights and terrain slope, were included in the final analysis for ensemble modelling.

#### Building the ensemble model

We fitted our data using seven model algorithms, namely generalised linear models (GLM), surface range envelopes (SRE), multivariate additive regression splines (MARS), artificial neural networks (ANN), BRT, also known as GBM (generalised boosted regression modelling), random forest (RF) and maximum entropy ecological niche models (MaxEnt). These algorithms are included within Biodiversity Modelling (BIOMOD) [48], a computational framework intended for modelling species distribution. BIOMOD was used to build the ecological niche model, and implemented with the package *biomod2* in R (v.3.3.2) [43]. The MaxEnt algorithm is not in-built in BIOMOD; however, there is provision to include this as an add-on. The software was developed by S. Phillips and colleagues and is freely available from https://biodiversityinformatics.amnh.org/open_source/maxent/ (v.3.4.1).

Ideally, model accuracy should be evaluated with data that are independent of the training data. As we did not have an independent dataset, the original data was partitioned into two, with a random sample of 30% of the original data retained for testing/evaluation and was considered ‘quasi-independent’, while the remaining 70% was used to train/calibrate the model. To evaluate the validity of model performance and accuracy on the quasi-independent data, BIOMOD offers an alternative ability to perform internal cross-validation whereby a set number of data splitting runs are computed. In each model run, the model is fitted to one part of the dataset and tested on the other part. This internal cross-validation does not provide a measure of predictive performance *per se*, but provides a measure of internal consistency of models [49]. We performed an iteration of 100 model runs for each of the seven algorithms, and the evaluation values of each run was stored and then averaged, to make the final result more robust. Model evaluation was performed based on the area under the receiver operating characteristic (ROC) curve and the Hanssen-Kuipers discriminant (also known as true skill statistic, TSS). TSS compares the number of correct predictions, minus predictions attributable to random guessing [50], taking into account both sensitivity and specificity. Its value ranges from -1 to +1, where +1 indicates perfect score, 0 indicates random performance and values of 0.5 or higher are generally considered to indicate acceptable model performance [49, 50]. The TSS value is not affected by the size of the validation dataset. Evaluation values for the cross-validation runs were then compared to the values from the runs from the quasi-independent data, checking for consistency in predictive accuracy scores.

The two best-performing model algorithms, based on ROC and TSS scores, were then selected for ensemble projection. The evaluation summaries of ensemble predictions are presented as mean ROC and TSS, median ROC and TSS, and lower and upper confidence bounds of ROC and TSS. Sensitivity and specificity were calculated and a threshold value that maximizes their sum (optimal threshold value for each condition) was considered to generate binary maps that display areas where LF transmission is more likely to occur based on environmental suitability.

A gridded map of estimated population density for Nigeria was obtained from the WorldPop Africa dataset [51]. Population density data available for Nigeria from this resource were for the years 2006, 2010, 2015 and 2020. As our data spanned from 2000–2013, we estimated the population based on population density estimates for the year 2010. We calculated population for each state by summing estimated numbers of people per pixel falling within predicted LF suitable areas and aggregating this to represent population by state. This analysis was performed using the Zonal Statistics function available within the Spatial Analyst Tool in ArcGIS 10.3 [52].

## Results

Analysis was performed using 1192 survey sites reporting presence-absence of LF covering all 36 states and the Federal Capital Territory in Nigeria. Survey participants were tested for the presence of filarial antigenemia using ICT. In total, 142,881 individuals were surveyed and 11,479 tested positive for LF infection.

**Fig. 2:**
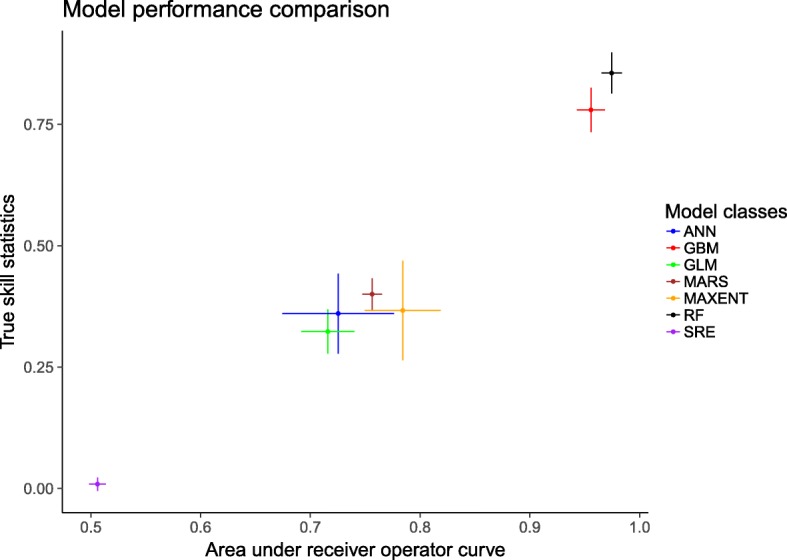
Model performance comparison by area under the receiver operating characteristic curve (ROC) and true skill statistic (TSS) values of all model classes. The points represent the mean estimates and the solid lines represent the 95% confidence intervals. *Abbreviations*: ANN, artificial neural networks; GBM, generalised boosted models; GLM, generalised linear models; MARS, multivariate additive regression splines; MAXENT, maximum entropy ecological niche models; RF, random forest; SRE, surface range envelope

Figure 2 shows the performance of the seven model algorithms implemented in the BIOMOD package. Here, the RF and GBM models outperform the others with area under the ROC and TSS values of > 0.95 and > 0.75, respectively. The two hundred models generated by these two algorithms where therefore chosen for constructing the final ensemble model.

**Fig. 3:**
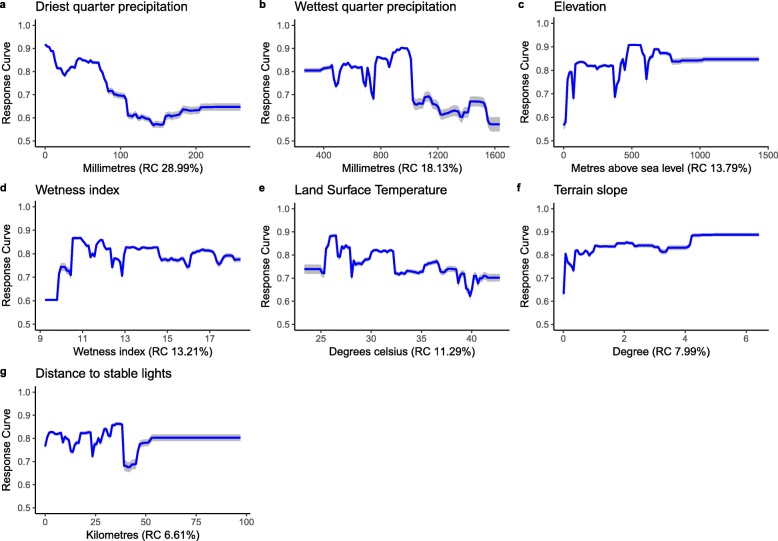
Marginal effects curves for covariates included in 100 ensembles of generalised boosted models. Blue lines represent the mean marginal effect and grey shading indicates the 95% bootstrap confidence intervals. The figures in parentheses indicate the relative contribution (RC) of each covariate, which add up to 100%. The Y-axis is the response (probability of LF occurrence) and the X-axis is the full range of covariate values

**Fig. 4:**
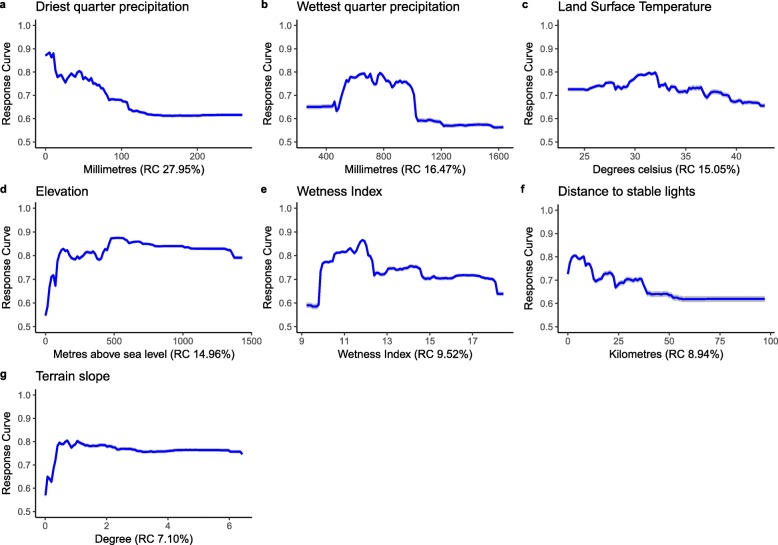
Marginal effects curves for covariates included in 100 ensembles of random forest models. Blue lines represent the mean marginal effect and grey shading indicates the 95% bootstrap confidence intervals. The figures in parentheses indicate the relative contribution (RC) of each covariate, which add up to 100%. The Y-axis is the response (probability of LF occurrence) and the X-axis is the full range of covariate values

Figures 3 and 4 show the relative contribution (RC, as percentage) and marginal effect plots of each covariate on the predicted suitability of occurrence for LF to the final GBM and RF models, respectively. For both model algorithms, precipitation of the driest quarter, precipitation of the wettest quarter, and elevation, were the major contributors to the ensemble of models. In total these three covariates contributed 60.91 and 59.38% to the fitted ensemble of GBM and RF models, respectively. The probability of LF occurrence appeared to steadily decrease with increasing precipitation of the driest quarter. High suitability for LF was positively associated with elevation up to 500 metres above sea level (masl), and then appeared to flatten up to 1500 masl. Land surface temperature and distance to stable lights also showed a negative correlation with LF occurrence.

A continuous risk map of environmental suitability of LF was projected on a geographical space based on the pre-selected environmental predictors (those shown in Figs. 3 and 4). The mean area under the ROC values on the evaluation dataset (30% of the full dataset) for the final ensemble model was 0.991 and the median was 0.993 (95% CI: 0.879–0.995). These areas under the ROC values measure the performance of the final ensemble model in fitting the presence-absence data and predicting across unsampled locations.

**Fig. 5:**
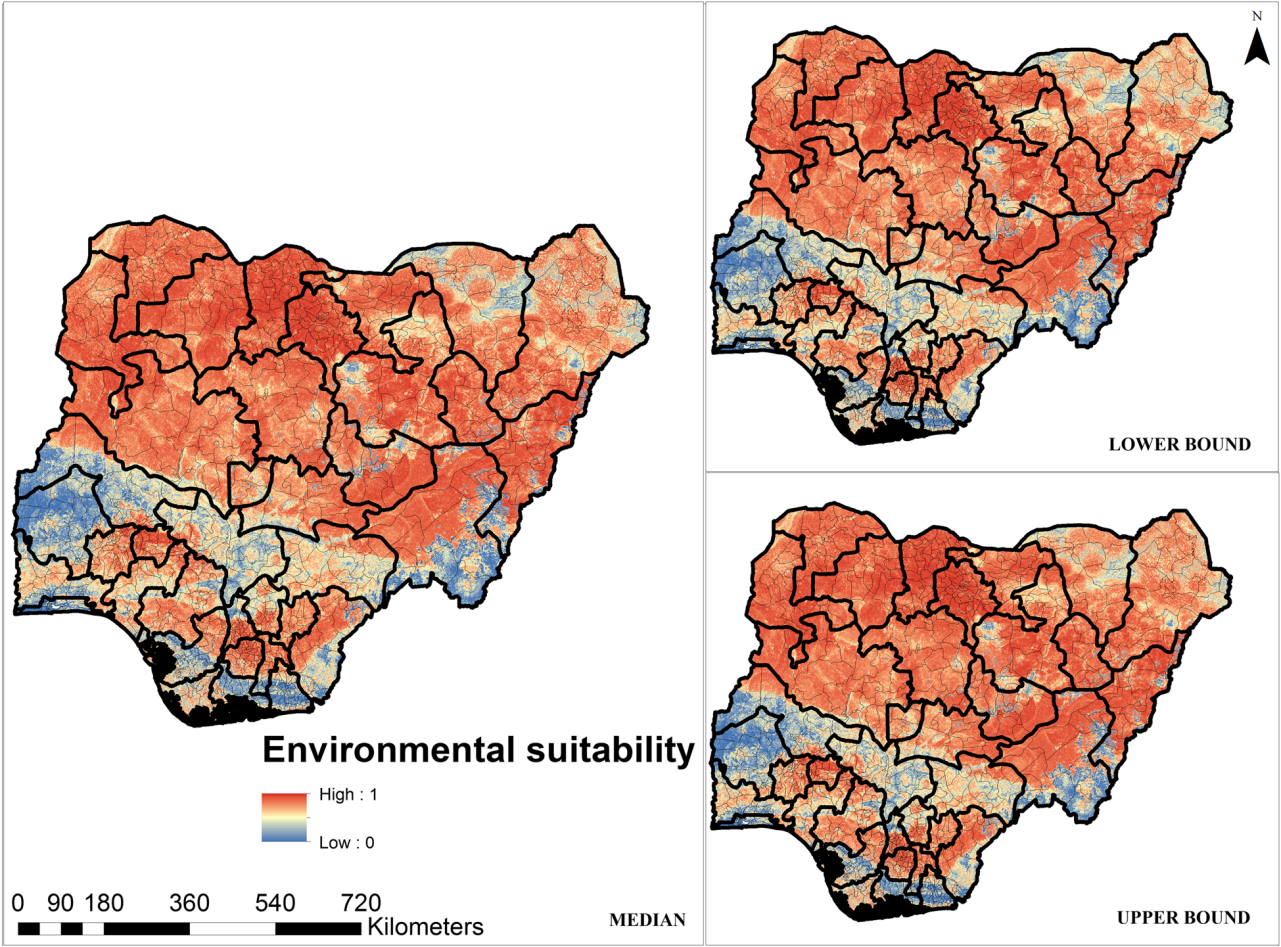
Median predicted environmental suitability of LF with the lower and upper bounds of the occurrence limits

The map presented in Fig. 5 suggests that large proportions of northern Nigeria are environmentally suitable and better able to drive LF transmission, although low suitability was predicted in the north-central state of Kogi. Low LF suitability was however predicted in most southern states in Nigeria.

**Fig. 6:**
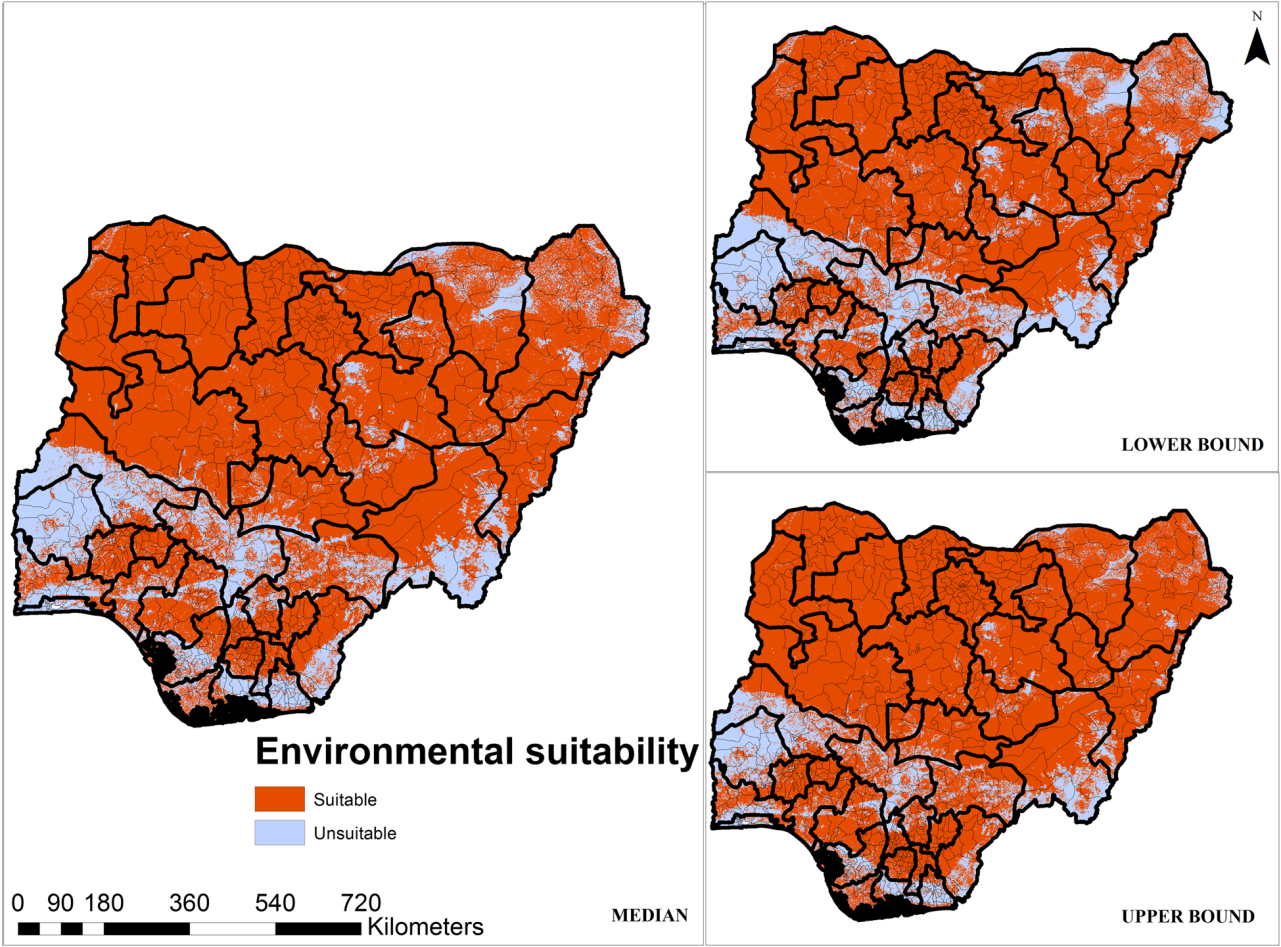
Median predicted binary map of environmental suitability for LF with the lower and upper bounds of the occurrence limits

A suitability threshold of 0.711 with a sensitivity of 95% and a specificity of 96.2% provided the best discrimination between presence and absence values according to the evaluation dataset, and therefore was used to reclassify the continuous predictive maps into binary maps, delineating land areas into either suitable or unsuitable for LF transmission (Fig. 6).

### Estimating population at risk

The total national population living in areas that are environmentally suitable for LF was estimated to be 110 (95% CI: 106–124) million, which corresponds to about 67% of Nigeria’s population in 2010. The largest portion of the population living in areas environmentally suited to LF transmission were found in Kano, Kaduna, and Katsina states with predicted populations of 9.6, 5.9 and 5.6 million, respectively (Table 2). All other states had population in at-risk areas of less than 5 million.

**Table 2:** Estimated human population living in areas environmentally suited to LF transmission by state in Nigeria in 2010

Zones in Nigeria		Population in areas environmentally suited to LF transmission	Total population
North-central States	Benue	2,997,209	4,853,000
	Kogi	1,299,057	3,838,000
	Kwara	841,730	2,852,000
	Nassarawa	2,007,317	2,151,000
	Niger	4,342,252	4,538,000
	Plateau	3,568,619	3,659,000
	FCT	1,438,127	1,537,000
Subtotal		16,494,311	23,428,000
North-east States	Adamawa	3,087,599	3,272,000
	Bauchi	4,893,787	5,257,000
	Borno	4,115,294	4,752,000
	Gombe	2,755,106	2,773,000
	Taraba	2,061,291	2,657,000
	Yobe	2,228,136	2,652,000
Subtotal		19,141,213	21,363,000
North-west States	Jigawa	4,701,572	5,054,000
	Kaduna	5,903,960	6,927,000
	Kano	9,625,825	10,765,000
	Katsina	5,640,911	6,550,000
	Kebbi	3,700,485	3,758,000
	Sokoto	3,973,503	4,137,000
	Zamfara	3,462,653	3,689,000
Subtotal		37,008,909	40,880,000
South-east States	Abia	2,034,246	3,269,000
	Anambra	4,314,081	4,819,000
	Ebonyi	2,251,489	2,345,000
	Enugu	2,778,413	3,717,000
	Imo	4,190,754	4,402,000
Subtotal		15,568,983	18,552,000
South-south States	Akwa Ibom	1,073,592	4,461,000
	Cross River	2,351,796	3,472,000
	Bayelsa	1,206,577	2,087,000
	Rivers	1,630,531	5,759,000
	Delta	2,025,928	4,747,000
	Edo	2,910,697	3,804,000
Subtotal		11,199,121	24,330,000
South-west States	Ekiti	2,357,067	2,516,000
	Lagos	538,364	14,480,000
	Ogun	1,281,100	3,953,000
	Ondo	2,608,852	3,679,000
	Osun	3,020,890	4,105,000
	Oyo	1,496,952	6,532,000
Subtotal		11,303,129	35,265,000
Sum total		110,715,856	163,818,000

## Discussion

In this study, we have produced maps at a resolution of 1 km^2^ to inform ongoing interventions by delineating areas of highest transmission risk which are prone to resurgence, thus to help in efficiently targeting control measures at the lowest administrative level. Our occurrence map (Fig. 6) indicates that suitability to LF transmission is widely distributed in Nigeria. However, parts of the north-east state of Borno, southern states of Cross River, Rivers, Akwa Ibom, Delta and Edo, and south-west states of Lagos, Oyo, Ogun and Ondo, are not environmentally suited to LF transmission. According to our estimates, about 67% of the Nigerian population live in areas environmentally suitable for LF transmission.

The benefits of machine learning algorithms compared to logistic regression models for niche and species distribution modelling have been thoroughly reviewed [45, 53–58]. Machine learning algorithms allow to account for complex non-linear associations between the response (e.g. disease occurrence) and explanatory variables, and control for interactions among explanatory variables [19]. Furthermore, combining predictions from more than one modelling algorithm to form an ensemble has been found to produce more precise estimates when used for ecological niche and species distribution models [59]. In our work, we have mapped the ecological niche of LF in Nigeria using an ensemble of two algorithms (GBM and RF), which are widely considered to produce accurate estimates of disease distribution [46, 58]. GBM models combine fitting regression trees with boosting, by recursively partitioning data into smaller binary splits, and splits repeatedly applied to their own until the best split is chosen [46]. ‘Boosting’ allows fine tuning of the overall model and is performed in a forward stepwise manner minimising residual variation in the response [46]. The combination of regression trees and boosting have been demonstrated to avoid over-fitting [45]. For RF models, a large number of trees are grown with the root node of each new tree containing a different random bootstrap prediction of the original data [58, 60]. For final predictions, the average values of all bootstrapped predictions are taken. It has been demonstrated that RF models are efficient in tuning and improving the accuracy of models [58].

Our work provides an insight into the regional distribution of LF in Nigeria, and we find that the areas less suitable for LF transmission correspond to mangrove ecosystems and freshwater swamps in the southern parts of the country, and also to short grass savanna in the north-east [61]. We have identified environmental factors associated with the occurrence of LF in Nigeria, with precipitation during the driest quarter contributing the most in driving the probability of LF occurrence. This finding shows that availability of temporal breeding sites during the driest period is critical for the major LF vectors, *Anopheles* spp. mosquitoes, to sustain the transmission [1]. However, marginal effect plots also showed that the probability of LF occurrence would decline when precipitation exceeds 800 mm during the wettest quarter of the year, which may suggest that although rainfall is required for vector survival and breeding, excessive rainfall may cause flooding and destruction of breeding sites [16, 19]. Similarly, the probability of LF occurrence started to decline at high land surface temperatures. This is consistent with experimental findings on adult mosquito survival and larval development [62–64], which suggest that both adults and larvae are unable to thrive at high temperatures.

The probability of LF occurrence appeared to increase with increasing elevation, and levels off at around 500 metres above sea level. This phenomenon has been previously recorded [16, 19] and is thought to reflect the negative effect of decreasing temperature with increasing altitude on mosquito survival and the rate of parasite development within the vector [65]. We found a negative correlation between higher terrain slope and suitability of environment to LF, perhaps because steeper inclinations of terrain cause more rapid surface water runoff, thus reducing the collection of water pockets which may serve as breeding sites for mosquito vectors.

Finally, it seems that environmental suitability for LF steadily declined with increasing distance from stable lights. Stable light is considered for any pixels at a value of > 0; however, values < 0 do not mean total pixel darkness but rather that the intensity of light emitted does not reach the threshold to be captured by the sensor. Here we took stable lights as proxy for rural-urban divide and economic activity, as urban areas are more likely to emit night-light (and thus stable lights) than rural areas. As the distance from stable night-light increased, the probability for LF occurrence decreased. This drop may be explained by the absence of stable lights in uninhabited areas where the mosquito population is more likely to be of low abundance, or in more rural settings where stable lights are less likely to be present, as electricity is in short supply in large parts of rural Nigeria. Although LF has always been associated with more rural areas [66–68], a recent study in Tanzania has highlighted the burden of LF in urban settings [69], and corroborated in a study conducted in an urban Nigerian setting [68]. Studies have also illustrated that mosquitoes are more likely to aggregate around human populations [70, 71]. Smith et al. [70] reported that the distribution of the human population influenced the aggregation of adult mosquitoes because mosquitoes are more likely to gravitate towards the human host. These authors demonstrated that mosquito density was lowest in rural settings but higher in peri-urban and urban settings.

In Nigeria, *Anopheles* spp*.* are the principal vectors for LF [1, 72, 73], and our maps of environmental suitability correspond well with known historical distribution patterns of these mosquitoes in Nigeria [1]. This may be due to the relatively stable nature of the peri-domestic environmental factors driving the abundance and distribution of *Anopheles* mosquitoes in the past 20 years [16]. Previous studies have demonstrated that environmental factors may affect mosquito species differently. For instance, precipitation, which was a major driving factor in our model, is thought to have a greater effect on *Anopheles* spp. than it does on *Culex* spp. [16], perhaps due to their different breeding habitats. *Culex* mosquitoes are known to breed in areas with poor sanitary and housing conditions [3], thus human factors may play a more important role than precipitation. It will therefore be interesting to test our model in geographical areas where *Culex* spp. are the predominant vectors for LF. Furthermore, although LF transmission has been interrupted in the north-central states of Plateau and Nasarawa [26], the mosquito vectors remain; thus there is a risk of recrudescence due to within-country human migration [51] and a possible re-introduction of the LF parasite.

Estimates of the human population living in areas environmentally suited to LF transmission have steadily increased over the years and varied significantly in previous studies [74–76]. This may be due to improved diagnosis and surveillance as well as population growth. In 1992, an estimated 113 million people lived in LF at-risk areas in Africa [74] and by 2009, this figure was estimated to be 212 million [76]. Nigeria was reported to have the third highest national LF burden with an estimated 114 million individuals living in at-risk areas in 2016 [15]. These estimates are usually calculated by summing up the populations of each of district where infection is detected, and may thus overestimate the actual LF burden since this approach does not account for within-district spatial variations and is highly dependent on the existence of field survey data in endemic areas. In addition, field surveys are more likely to be carried out where LF infection is suspected or in areas where universities are located [77] and thus surveillance may in some locations fail to capture the true infectious status of vast areas of the country. Since 2000, Nigeria has seen an increase in violence and militancy in the southern Niger Delta regions [78], and terrorist activities in the north-eastern parts of the country [78, 79]. These conditions make it difficult to carry out field surveys and data from these areas are patchy. In contrast, estimates of the human population at risk derived by geostatistical and machine learning-based models estimate the complete distribution of infection and thus may produce more accurate estimates of the true extent of infection and the human populations at risk [19]. The additional prospect of our model producing a threshold value that maximises the sum of specificity and sensitivity improves the accuracy of our binary map (Fig. 6) [48, 80].

The human population living in areas classified as environmentally suitable for LF transmission in Nigeria using a machine learning approach was previously estimated to be 143.97 million [19]. This figure was derived from modelling only 27 presence-only data points from surveys conducted between 1977 and 1990 and of varying diagnostic methods and study designs, whereas our model was implemented using 1192 presence-absence data points derived from standardised field surveys. Furthermore, niche models derived by using presence-only data points are often spatially biased as surveys are usually conducted in areas that are more easily accessible, and are usually positive (presence) counts [81]. This bias is usually remedied by selecting background or pseudo-absence data points [82], that is assumed absence data drawn at random for the region of interest, to balance the presence-absence data point ratio. Since presence points are usually concentrated in particular geographical regions due to convenience of sampling, by randomly selecting pseudo-absence data points analysis may be biased as true presence points might be treated as ‘absence’, and thus leading to inaccurate model predictions [81]. The geographical spread and the amount of presence-absence data used in the present study, together with the standardised methods for field surveys and data collection, implies that our models provide accurate population estimates of the number of individuals living in areas environmentally suited to LF transmission.

## Conclusions

The data used in this analysis represent a unique resource and provide the most comprehensive database for LF distribution in Nigeria. As the national LF control programme moves towards elimination, the methods and results presented in this study will inform surveillance activities and help optimise resource allocation for disease control.

## Abbreviations

AfSIS: African Soil Information System
ANN: Artificial neural networks
BIOMOD: Biodiversity modelling
BRT: Boosted regression trees
CI: Confidence interval
DEM: Digital elevation model
ENM: Ecological niche model
EVI: Enhanced vegetation index
GBM: Generalised boosted models
GLM: Generalised linear models
ICT: Immunochromatographic card tests
ISRIC: Soil Reference and Information Centre
IU: Implementation unit
LF: Lymphatic filariasis
LGA: Local government area
LST: Land surface temperature
MARS: Multivariate additive regression splines
MaxEnt: Maximum entropy ecological niche models
MDA: Mass drug administration
MERIS: Medium resolution imaging spectrometer
MODIS: Moderate resolution imaging spectoradiometer
NL: Night-light
NLFEP: Nigerian Lymphatic Filariasis Elimination Programme
PET: Potential evapo-transpiration
RC: Relative contribution
RF: Random Forest
ROC: Receiver operating characteristic curve
SCA: Specific catchment area
SRE: Surface range envelopes
SRTM: Shuttle Radar Topography Mission
TAS: Transmission assessment survey
TSS: True skill statistic
TWI: Topographic wetness index
VIF: Variance inflation factor
WHO: World Health Organization
